# Temporal Dynamics of Solid-State Thermally Activated Delayed Fluorescence: Disorder or Ultraslow Solvation?

**DOI:** 10.1021/acs.jpclett.1c03810

**Published:** 2022-02-17

**Authors:** Tomas Serevičius, Rokas Skaisgiris, Jelena Dodonova, Irina Fiodorova, Kristijonas Genevičius, Sigitas Tumkevičius, Karolis Kazlauskas, Saulius Juršėnas

**Affiliations:** † Institute of Photonics and Nanotechnology, Vilnius University, Saulėtekio 3, LT-10257 Vilnius, Lithuania; ‡ Institute of Chemistry, Vilnius University, Naugarduko 24, LT-03225 Vilnius, Lithuania; § Institute of Chemical Physics, Vilnius University, Saulėtekio 3, LT-10257 Vilnius, Lithuania

## Abstract

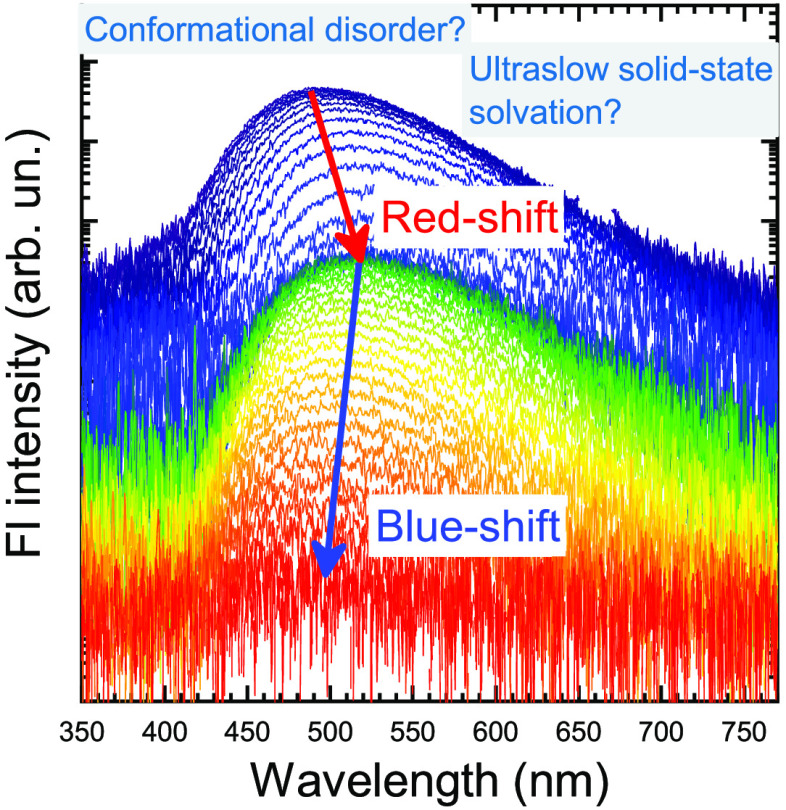

Time-resolved emission spectra of thermally activated delayed fluorescence (TADF) compounds in solid hosts demonstrate significant temporal shifts. To explain the shifts, two possible mechanisms were suggested, namely, slow solid-state solvation and conformational disorder. Here we employ solid hosts with controllable polarity for analysis of the temporal dynamics of TADF. We show that temporal fluorescence shifts are independent of the dielectric constant of the solid film; however, these shifts evidently depend on the structural parameters of both the host and the TADF dopant. A ≤50% smaller emission peak shift was observed in more rigid polymer host polystyrene than in poly(methyl methacrylate). The obtained results imply that both the host and the dopant should be as rigid as possible to minimize fluorescence instability.

The most efficient organic light-emitting diode (OLED) devices are made of emissive thermally activated delayed fluorescence (TADF) compounds.^1−3^ The high emission yield of TADF compounds is achieved by minimizing the singlet–triplet energy gaps (Δ*E*_ST_) and enabling thermally activated reverse intersystem crossing of triplet states.^4,5^ Among the approaches that are suggested to enable TADF, like the multiresonance effect^6^ or hot-exciton pathway,^7^ the most popular is based on constructing TADF compounds from electron-donating (D) and electron-accepting (A) fragments.^8^ In this case, a lower Δ*E*_ST_ is achieved by minimizing the electron exchange energy, which in fact depends on the HOMO–LUMO overlap.^9^ Therefore, building the molecular compounds of D and A units decouples HOMO and LUMO and enables triplet upconversion.^10^ The efficient separation of frontier molecular orbitals could be achieved either by twisting D and A units toward orthogonality^11^ or by keeping the molecular structure rather flat but with extended conjugation in the HOMO and LUMO.^12,13^ Despite the chosen design strategy, TADF compounds are still composed of singly bonded molecular units with high twisting flexibility and pronounced charge-transfer character. TADF decay processes in solutions are rather straightforward; however, in the solid state, they are accompanied by temporal spectral shifts and increased delayed fluorescence lifetimes,^14−19^ which is disadvantageous for OLEDs, as it accelerates efficiency roll-off at large luminance values.^20^ To date, two possible mechanisms have been proposed to explain the temporal dynamics of solid-state TADF. The first relies on conformational disorder^14^ and suggests that the solid surrounding locks the TADF structures in a variety of D–A twisting conformations, having different singlet–triplet energy gaps^14−19^ and, therefore, different radiative decay rates (*k*_r_) and emission wavelengths. In the case of prompt fluorescence (PF), initial decay occurs from the conformer states with the largest *k*_r_ and thus the largest singlet energy. In contrast, the later decay is associated with conformers with a lower *k*_r_ and a smaller singlet energy, hence enabling the red-shift of prompt fluorescence (PF). In the case of delayed fluorescence (DF), the trend is the opposite as the molecular conformations with the lowest radiative decay rate (stronger charge-transfer character) will have the smallest Δ*E*_ST_ and therefore the most rapid DF, enabling its blue-shift. Moreover, DF properties tend to be strongly dependent on the TADF dopant concentration due to the selective concentration quenching of long-lived triplet species.^21^ Solid-state solvation^22−25^ was suggested as the second mechanism responsible for temporal DF shifts. From this point of view, the stabilization of excited TADF molecules is ultraslow due to the hindered dipole orientation of the host matrix, enabling temporal shifts of emission up to milliseconds. It is noteworthy that larger emission shifts are typically found in more polar solid hosts providing stronger dipole–dipole interaction between the dopant and host.^24^ On the contrary, there are experimental and numerical reports stating that slow solid-state solvation (SSS) is actually ultrafast and, thus, cannot explain the temporal dynamics of TADF in the solid state.^14,26^ Therefore, knowing the actual mechanism is crucial, as the increase in emission yield is usually achieved by embedding TADF dopants in polar hosts, fostering the decrease in Δ*E*_ST_.^25^

To date, most of the studies analyzing the temporal behavior of solid-state TADF are performed in different hosts, based on either polymer or small-molecule compounds.^15,22,24^ However, various host materials differ in more than one parameter. For example, although DPEPO {bis[2-(diphenylphosphino)phenyl]ether oxide} and PYD2 [2,6-bis(9*H*-carbazol-9-yl)pyridine] are two widely used hosts with different dielectric constants (ε),^14^ a simple comparison of emission properties in those hosts cannot discern the polarity^15^ from disorder effects.^24^ This is because compounds have completely different molecular structures, implying their different rigidities. DPEPO is thought to be a less rigid host than PYD2.^14^ In such a case, the less restrictive environment of DPEPO could enable greater conformational disorder causing larger temporal shifts of DF as compared to the shifts imposed by polarity. Therefore, it is important to discern the impact of polarity and rigidity and analyze both processes independently.

In this paper, we present a brief approach to the spectroscopic analysis of individual solvation and disorder effects in the solid-state TADF. Two polymer hosts with different molecular structures, polystyrene (PS) and poly(methyl methacrylate) (PMMA), were selected and later selectively doped with camphoric anhydride (CA) having a large ground-state dipole moment^26−29^ (see Figure 1). This allowed broad-range tuning of the ε values of PS and PMMA hosts.^26^ The molecular alignment of CA is more rapid than that of the polymer host,^24,26^ though both should contribute to the solvation. More pronounced interaction between CA and polar units in the polymer backbone is expected at larger CA doping loads through the dipolar communication, later leading to altered dipolar interactions between the excited TADF dopant and the ground-state host. Such subtle variations should also affect the PF shifts. In this study, **PXZ-PYR**(30) and **ACRPyr**(31) compounds possessing more and less flexible molecular structures, respectively, were selected as TADF emitters. The comparison of emission properties of these emitters in the same host but featuring different ε values allowed us to visualize the polarity effects; meanwhile, the disorder effects were assessed by comparing the temporal dynamics of TADF compounds dispersed in hosts with the same polarity but a different molecular structure.

**Figure 1:**
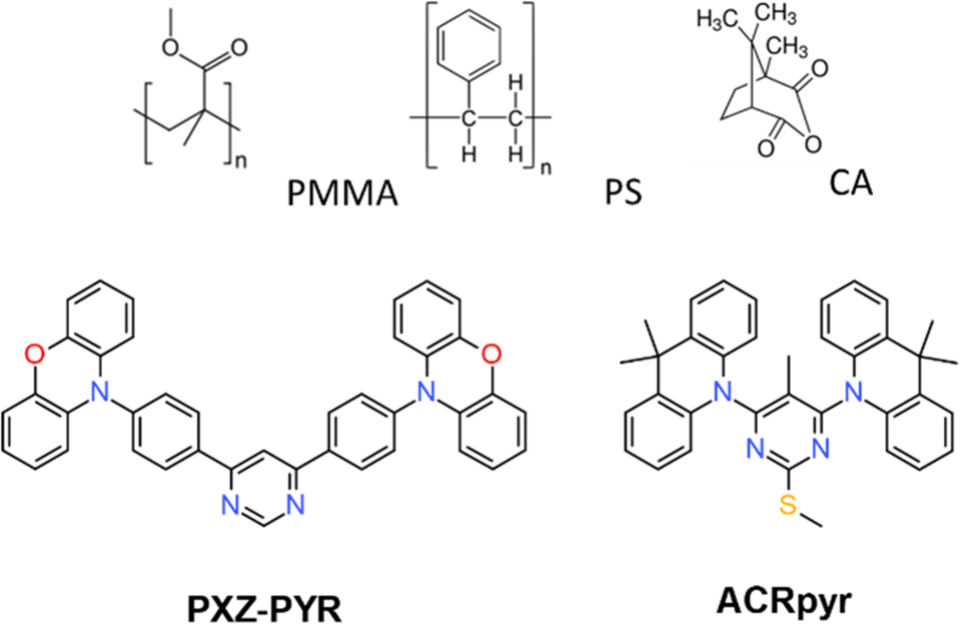
Molecular structures of PMMA, PS, CA, **PXZ-PYR**, and **ACRPyr**.

At first, a solvatochromic study of the compound solutions was performed (see Figure 2). Typical behavior for charge-transfer (CT) compounds was observed, namely, the red-shift of the emission peak and an increase in the full width at half-maximum (fwhm).^32^ Remarkably stronger solvatochromic behavior was observed for **PXZ-PYR** with an ∼680 meV red-shift, compared to an ∼220 meV shift for **ACRPyr** when utilizing a solvent with increasing polarity [from cyclohexane to tetrahydrofuran (THF)]. To assess the controllable solvation in the solid state, camphoric anhydride was employed. A high ground-state dipole moment with a small CA molecule (∼6 D^27^) enabled the selective tuning of the polarity of polymer films when doped with CA at different loads^26−29^ (see Figure 3a). As one can see, the dielectric constant of polymer films can be tuned in a broad range of 2.45–4.97 for PS (same as in the report by Madigan et al.^27^) and 3.41–8.31 for PMMA with a linear ε dependence on CA doping load in the range of 0–20 wt % (see also Table S1). CA was transparent up to nearly 260 nm, being optically inactive in the wavelength range of **PXZ-PYR** and **ACRPyr** (see Figure S1). No traces of any aggregate species were observed in the absorption spectra of **PXZ-PYR** and **ACRPyr** in PS and PMMA hosts doped with CA (see Figure S2).

**Figure 2:**
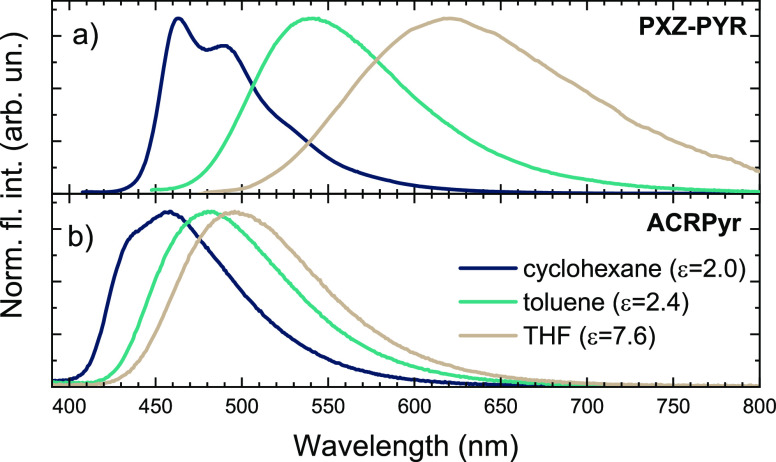
Fluorescence spectra of **PXZ–PYR** and **ACRPyr** in dilute cyclohexane, toluene, and THF solutions.

**Figure 3:**
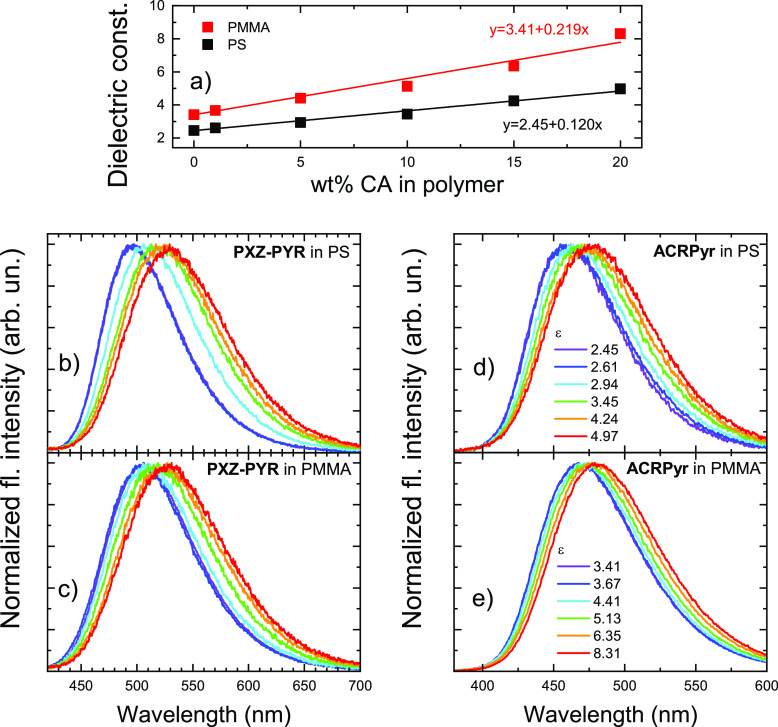
(a) Dielectric constants of PS (black) and PMMA (red) films with different CA doping loads. Color lines are linear fits. Fluorescence spectra of **PXZ-PYR** and **ACRPyr** in PS (b and d, respectively) and PMMA (c and e, respectively) films with different dielectric constants (different CA doping loads). The concentration of the TADF dopant was set at 1 wt %.

Fluorescence spectra of **PXZ-PYR** and **ACRPyr** in PS and PMMA films with different CA doping loads are shown in Figure 3b–e. Like solutions, typical positive solvatochromic behavior was observed for emission spectra (see Table S2 for details). Emission peak shifts of 150 and 110 meV for **PXZ-PYR** and 100 and 70 meV for **ACRPyr** in PS and PMMA films, respectively, were observed. fwhm values of emission spectra followed the same trend, systematically increasing for 15% or 7% for **PXZ-PYR** and 12% or 2% for **ACRPyr** in PS and PMMA films. The solvatochromic emission shift of **PXZ-PYR** was like that of **2PXZ-OXD**,^28^ a TADF dopant with a similar molecular structure (∼150 meV for the CA doping range of 0–20 wt %). SSS behavior was weaker than in solutions due to the more restrictive solid surrounding.^28^ As in solutions, **ACRPyr** showed a smaller shift of the emission peak with an increase in ε, most likely due to the lower excited-state dipole moment.^14^ This was also evident from Lippert–Mataga plots of **PXZ-PYR** and **ACRPyr** in PS and PMMA films, relating the Stokes shift and the change in the dipole moment upon photoexcitation (see Figure S3). Such a positive SSS course implies stronger stabilization of the excited state in a more polar surrounding. Therefore, if dipole–dipole interactions between the host and dopant are the driving force of slow temporal emission dynamics, increasing the dielectric constant of a polymer film should alter the temporal fluorescence shifts.^22,24^

To test this assumption, time-resolved fluorescence spectra of **PXZ-PYR** and **ACRPyr** were measured in PS and PMMA films with additional CA doping (see Figure S4) and energy shifts were estimated (see Figure 4). The magnitude of the energy shift (Δ*E*) was described as the difference in the spectral onsets of the initial PF spectrum (at a 1.25 ns delay) and the latest PF spectrum (at an ∼100 ns delay)^16^ (see Figures S4 and S5). No evident changes in Δ*E* were found upon the increase in the level of CA doping (see Figure 4b,c), though it can be suggested that solvation may not be the driving force of temporal PL shifts. Δ*E* values were nicely scattered around the average energy shift value (Δ*E*_avg_). Although Δ*E*_avg_ was independent of the dielectric constant of the solid host, a clear correlation between the magnitude of the energy shift and the structural properties of the polymer and TADF dopant was observed. Such behavior, on the contrary, can be explained by a conformational disorder concept.^14−18,31^ Δ*E* clearly was lower for **ACRPyr** than for **PXZ-PYR** (144 meV vs 68 meV and 99 meV vs 55 meV in PMMA and PS, respectively). The main difference in molecular structure between **PXZ-PYR** and **ACRPyr** is the rotational flexibility of the donor units.^16^ Acridine in **ACRPyr** is sterically constrained by the 5-methyl unit with limited ability to rotate along the C–N axis.^31^ In the solid state, such a limited distribution of D–A twisting angles leads to a low dispersion of singlet-state energies, singlet–triplet energy gaps, and, therefore, small PF and DF peak shifts. In the case of **PXZ-PYR**, the phenoxazine donor unit is confined by only hydrogens in the spacing phenyl unit, leading to evidently greater rotational flexibility.^30^ Here, flexible **PXZ-PYR** has a wider distribution of S_1_ energies, leading to more pronounced temporal emission peak shifts. Moreover, Δ*E* was evidently smaller in PS films for both compounds, particularly for **PXZ-PYR** (144 meV vs 99 meV and 68 meV vs 55 meV for **PXZ-PYR** and **ACRPyr**, respectively). The conformational disorder (described by the magnitude of Δ*E*) is also controlled by the host parameters.^14^ A more rigid host has a more tightly packed environment with a reduced D–A twisting angle distribution. Therefore, in this case, PS could be regarded as more rigid polymer host, probably due to the presence of a rigid phenyl unit rather than a flexible ester fragment in PMMA; however, the exact nature is still unclear and needs more in-depth analysis. The combination of a rigid molecular structure and a rigid polymer host implied minimized conformational disorder for **ACRPyr** in PS with a Δ*E* of only 55 meV. Further reduction of spectral instability probably could be achieved by selecting even more rigid hosts, like well-known OLED host PYD2.^14^

**Figure 4:**
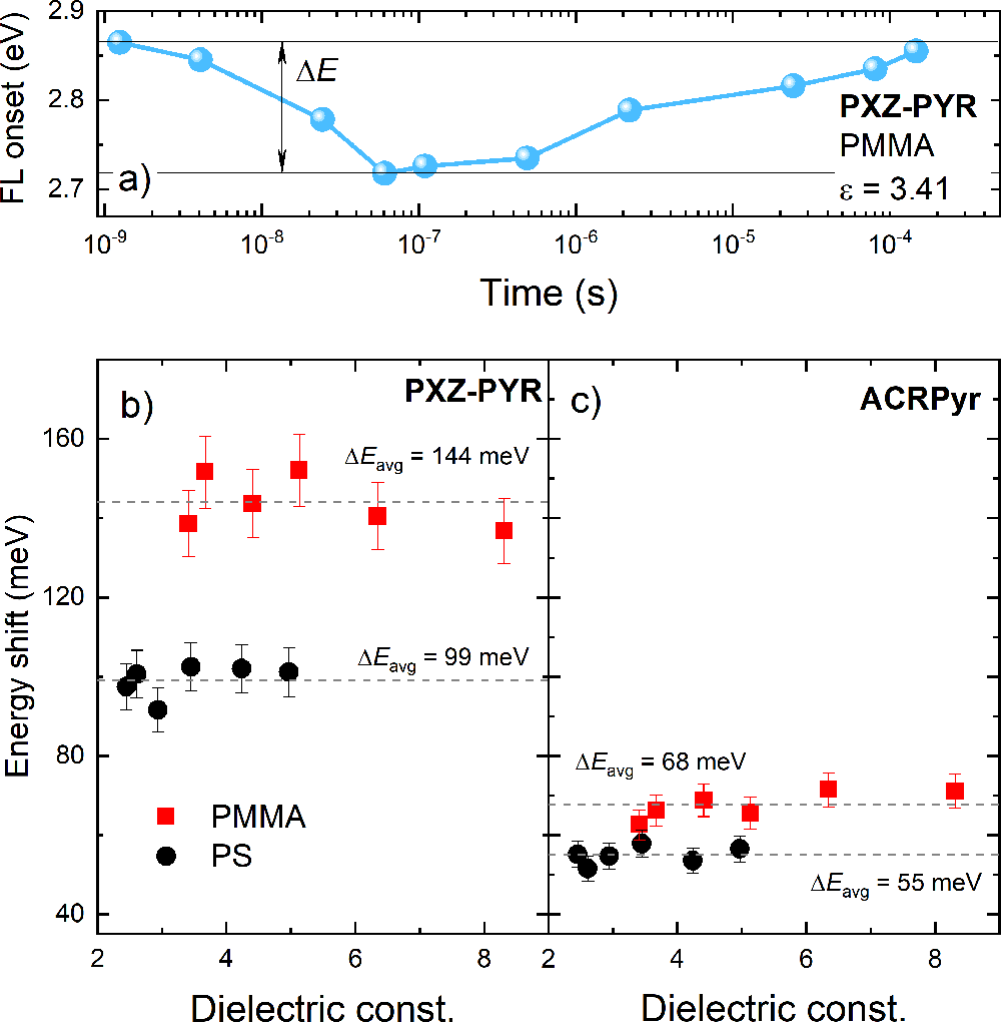
(a) Temporal dynamics of **PXZ-PYR** in a PMMA film with a 0 wt % CA doping load. Δ*E* shows the energy shift of PF.^16^ Energy shifts for (b) **PXZ-PYR** and (c) **PXZ-PYR** in PS (black) and PMMA (red) films with different ε values. Error bars are also included.

In conclusion, seeking to reveal the solvation role in the temporal emission dynamics, we performed the photophysical analysis of TADF compounds doped in two different polymer hosts with selectively tuned polarity. No evident dependence of the temporal shifts of prompt and delayed fluorescence on the dielectric constant of the polymer film was found. If solid-state solvation is the driving force of temporal emission dynamics, somewhat different shifts should have been observed in a more polar surrounding. However, the observed behavior was in accordance with a conformational disorder mechanism. Explicitly, a more sterically restricted molecular structure of the **ACRPyr** dopant favored smaller emission shifts, which were further diminished by employing a more rigid polymer host. Such enhanced spectral stability of TADF compounds is highly preferable for rapid triplet upconversion. For further proof of this mechanism, in-depth solvation studies in a more diverse solid surrounding are necessary. We believe our findings will improve our better understanding of the design concepts of TADF compounds and foster efficiency roll-off optimization in OLED devices.

## Experimental Methods

Synthesis and characterization of **PXZ-PYR** and **ACRPyr** were described previously.^30,33,34^ Photophysical properties of **PXZ-PYR** and **ACRPyr** were analyzed in PS and PMMA (Sigma-Aldrich) films at a concentration of 1 wt %. Solid-state films were prepared by dissolving each material at the appropriate ratio in toluene solutions and then wet-casting the solutions on quartz substrates in air. The dielectric constant of the polymer host was altered by adding various amounts of (±)-camphoric anhydride (CA)^28,29^ (Fine Synthesis Ltd.). Comparable pore radii of both polymers allowed us to compare SSS effects after doping with CA.^26^ The dielectric permittivity was estimated using the CELIV technique.^35^ Linearly increasing the voltage pulse was applied to neat and doped ITO/PS/Al (thickness of PS *d* = 200 nm) and ITO/PMMA/Al (thickness of PMMA *d* = 300 nm) sandwich structures. The thickness of the polymer film was estimated via a Dimension Icon atomic force microscope (Bruker). Initial current density step *j*_0_ was measured at a voltage rise speed *A* of 1 V/10 ms. The dielectric permittivity of polymer films was estimated according to equation ε = *j*_0_*d*/*Aε*_0_. Time-integrated and time-resolved fluorescence spectra were recorded using a nanosecond YAG:Nd^3+^ laser NT 242 (Ekspla, τ = 6 ns, pulse energy of 200 μJ, repetition rate of 1 kHz) and time-gated iCCD camera New iStar DH340T (Andor). Fluorescence decay transients were obtained by exponentially increasing the delay and integration time.^36^ The emission intensity was obtained by integrating all emission spectra. Solid-state samples were mounted in a closed cycle He cryostat (Cryo Industries 204N) for all measurements seeking oxygen-free conditions.

## Supporting Information Available

CA absorption spectrum, absorption spectra of **PXZ-PYR** and **ACRPyr** at different CA doping loads, Lipper–Mataga plots, and time-resolved PL spectra (PDF)
