# Supplementary material for: Temporal Dynamics of Solid-State Thermally Activated Delayed Fluorescence: Disorder or Ultraslow Solvation?
Source: J Phys Chem Lett. 2022 Feb 17;13(7):1839–44. doi: 10.1021/acs.jpclett.1c03810 (PMC8883520; doi:10.1021/acs.jpclett.1c03810)
Supplement: Supplementary file 1 — jz1c03810_si_001.pdf [file jz1c03810_si_001.pdf]

Supporting Information

Temporal Dynamics of Solid-State Thermally Activated Delayed Fluorescence: Disorder or Ultraslow Solvation?

Tomas Serevičius[a], Rokas Skaisgiris[a], Jelena Dodonova[b], Irina Fiodorova[b], Kristijonas Genevičius[c], Sigitas Tumkevičius[b], Karolis Kazlauskas[a] and Saulius Juršėnas[a]

[a]Institute of Photonics and Nanotechnology, Vilnius University, Saulėtekio 3, LT-10257 Vilnius, Lithuania.
[b]Institute of Chemistry Vilnius University, Naugarduko 24, LT-03225, Vilnius, Lithuania.
[c]Institute of Chemical Physics, Vilnius University, Saulėtekio 3, LT-10257 Vilnius, Lithuania.
*tomas.serevicius@tmi.vu.lt

Table S1 Dielectric constant of PS and PMMA films with different CA concentration. ε values of doped PS were alike as those in report of Madigan *et. al.*[1]

	Dielectric constant	
wt% CA	**PS**	**PMMA**
0	2.45	3.41
1	2.61	3.67
5	2.94	4.41
10	3.45	5.13
15	4.24	6.35
20	4.97	8.31

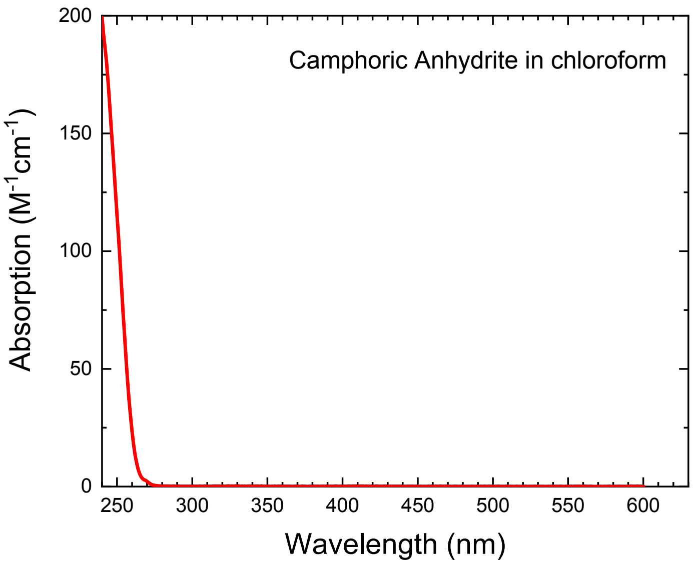

Fig. S1 Absorption spectrum of camphoric anhydride in chloroform. No absorption in the active range of **PXZ-PYR** and **ACRPyr** was found.

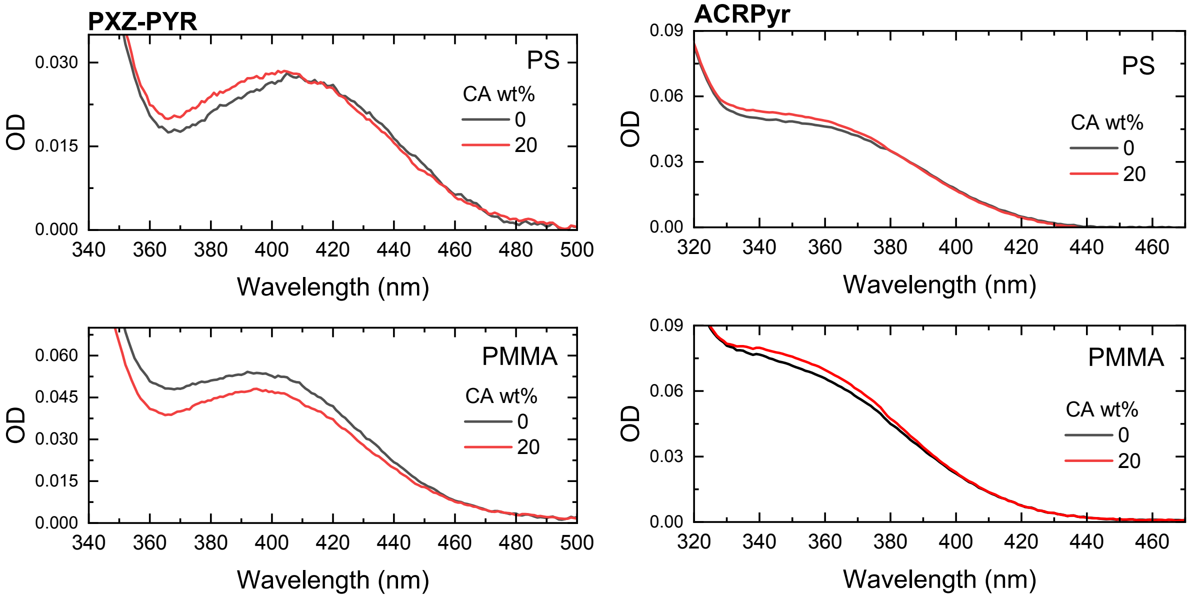

Fig. S2 Absorption spectra of **PXZ-PYR** (left picture) and **ACRPyr** (right picture) in PS and PMMA at 0 wt% and 20 wt% doping load of CA.

Table S2 Fluorescence peak values of **PXZ-PYR** and **ACRPyr** in PS and PMMA with different dielectric constant.

PS			*PMMA*		
	**PXZ-PYR**	**ACRPyr**		**PXZ-PYR**	**ACRPyr**
ε	E_{PL} (eV)	E_{PL} (eV)	ε	E_{PL} (eV)	E_{PL} (eV)
2.45	2.50	2.71	3.41	2.46	2.66
2.61	2.50	2.71	3.67	2.46	2.66
2.94	2.44	2.69	4.41	2.44	2.64
3.45	2.39	2.66	5.13	2.42	2.63
4.24	2.38	2.63	6.35	2.37	2.61
4.97	2.35	2.60	8.31	2.36	2.59

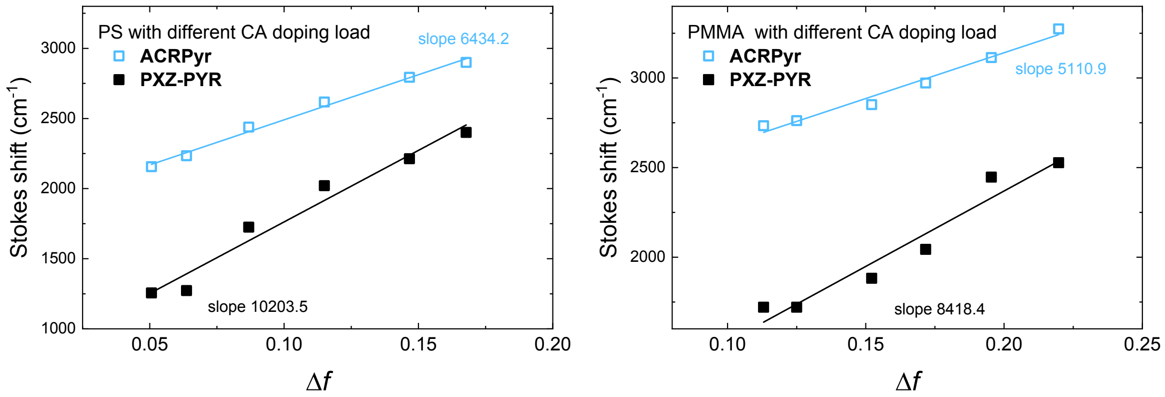

Fig. S3 Lippert-Mataga plots of **PXZ-PYR** and **ACRPyr** in PS and PMMA with different dielectric constant. Parameters were estimated according to Sk *et. al.*[2]. Refractive index (*n*) of polymer films with different CA concentration was taken as 1.4 for all doping loads for both polymers for simplicity. Any possible variation of *n* is much smaller than change in Stokes shift. Absorption spectra, according to Fig. S3 (ESI), were treated as independent on CA doping load. On-set values were taken for absorption energy calculations, while for emission energy estimation peak values were used.

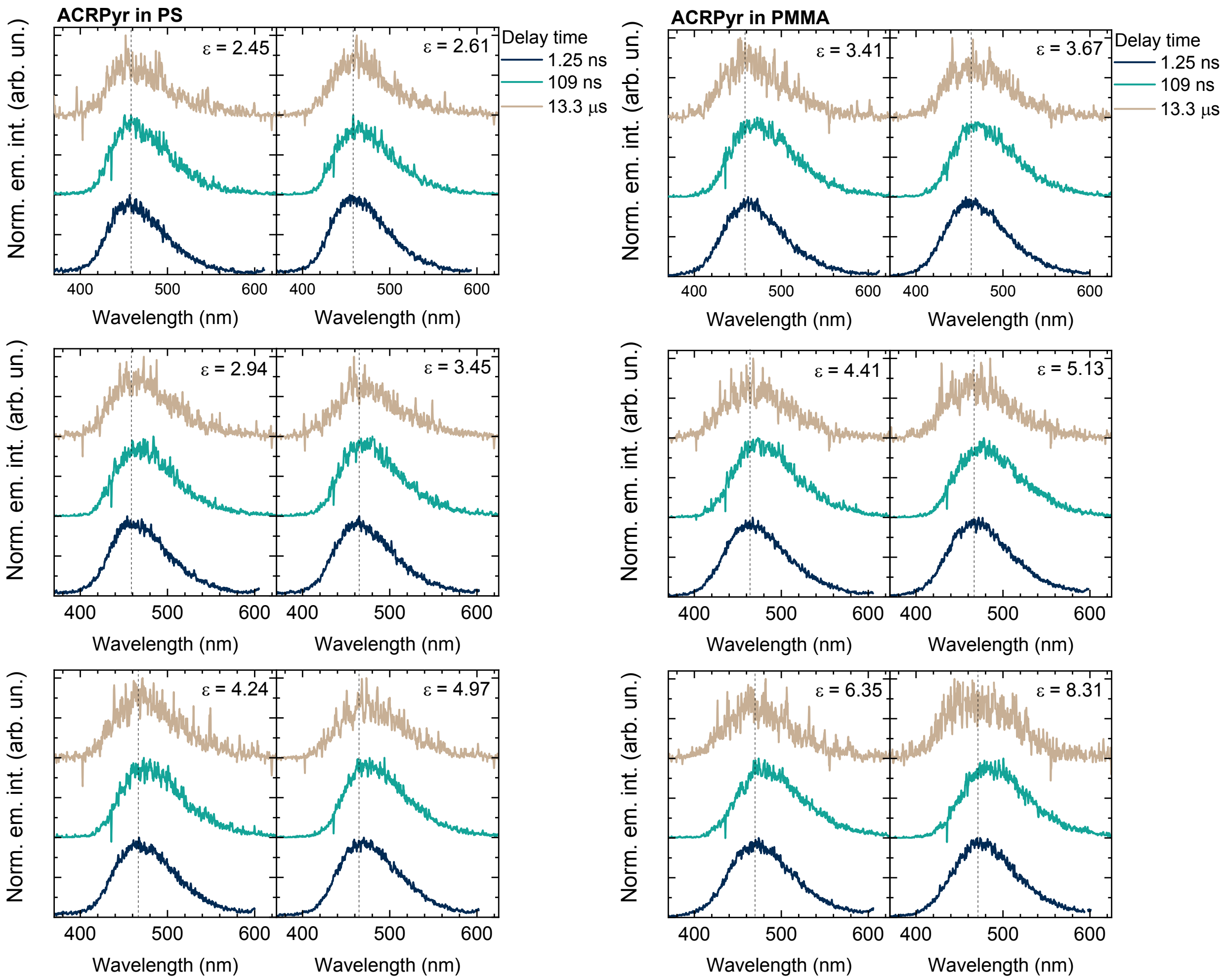

Fig. S4 Normalized time-resolved fluorescence spectra of **ACRPyr** in PS (right figures) and PMMA (left figures) with different dielectric constant. Spectra were shifted vertically for clarity.

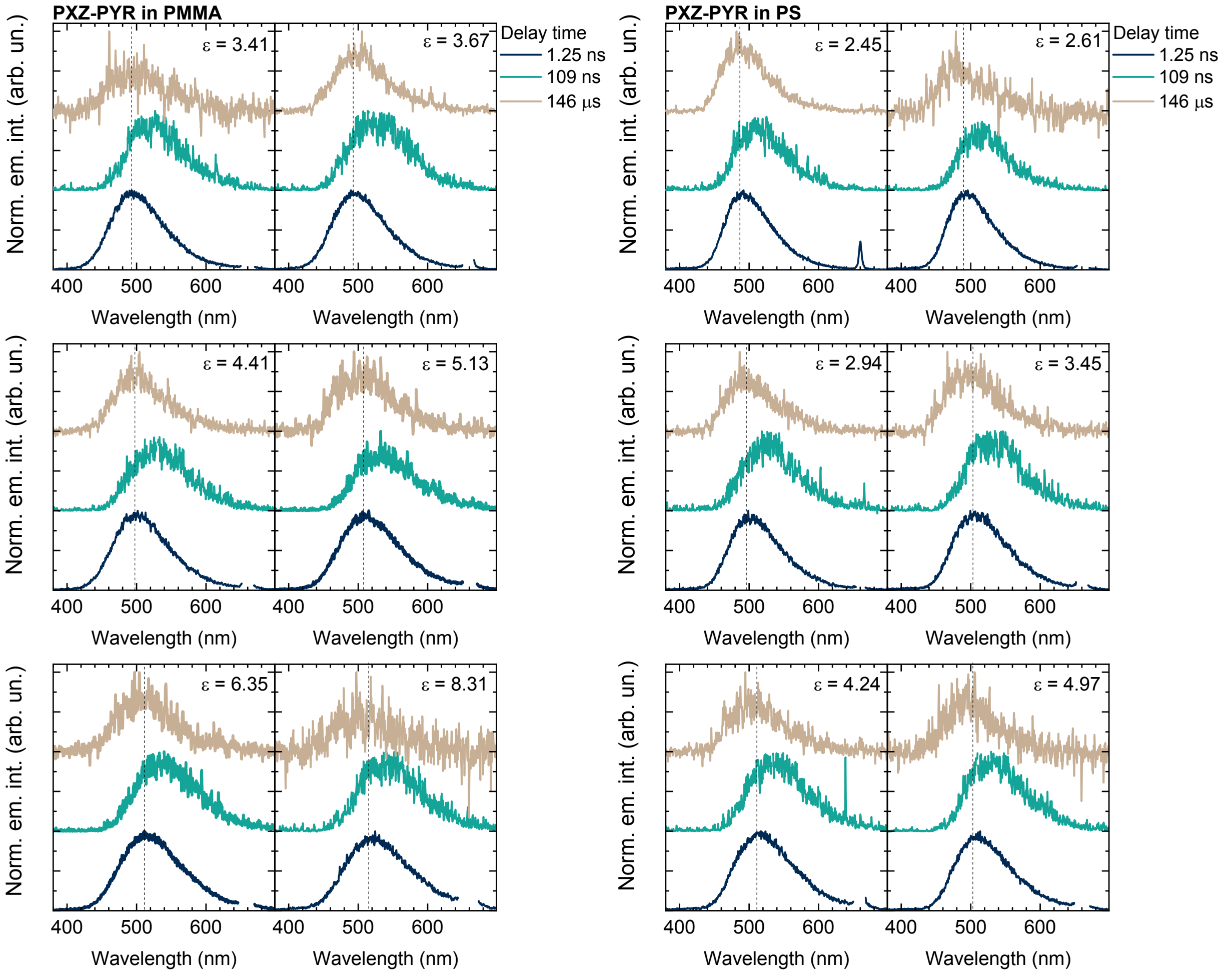

Fig. S5 Normalized time-resolved fluorescence spectra of **PXZ-PYR** in PS (right figures) and PMMA (left figures) with different dielectric constant. Spectra were shifted vertically for clarity.

References

(1) Madigan, C. F.; Bulović, V. Solid State Solvation in Amorphous Organic Thin Films. *Phys. Rev. Lett.* **2003**, *91* (24), 247403.

(2) Sk, B.; Thangaraji, V.; Yadav, N.; Nanda, G. P.; Das, S.; Parthasarathy, G.; Zysman-Colman, E.; Rajamalli, P. High Performance Non-Doped Green Organic Light Emitting Diode via Delayed Fluorescence. *J. Mater. Chem. C* **2021**, *9* (43), 15583–15590.
